# Mutation Rate and Effective Population Size of the Model Cooperative Bacterium *Myxococcus xanthus*

**DOI:** 10.1093/gbe/evae066

**Published:** 2024-03-25

**Authors:** Sébastien Wielgoss, James David Van Dyken, Gregory J Velicer

**Affiliations:** Department of Environmental Systems Science, Institute of Integrative Biology, ETH Zürich, 8092 Zürich, Switzerland; Department of Biology, Indiana University, Bloomington, IN 47405, USA; Department of Biology, University of Miami, Coral Gables, FL 33146, USA; Department of Environmental Systems Science, Institute of Integrative Biology, ETH Zürich, 8092 Zürich, Switzerland; Department of Biology, Indiana University, Bloomington, IN 47405, USA

**Keywords:** mutation rate (*µ*), effective population size (*N_e_*), myxobacteria, *Myxococcota*, sociality, mutation accumulation experiment

## Abstract

Intrinsic rates of genetic mutation have diverged greatly across taxa and exhibit statistical associations with several other parameters and features. These include effective population size (*N_e_*), genome size, and gametic multicellularity, with the latter being associated with both increased mutation rates and decreased effective population sizes. However, data sufficient to test for possible relationships between microbial multicellularity and mutation rate (*µ*) are lacking. Here, we report estimates of two key population-genetic parameters, *N_e_* and *µ*, for *Myxococcus xanthus*, a bacterial model organism for the study of aggregative multicellular development, predation, and social swarming. To estimate *µ*, we conducted an ∼400-day mutation accumulation experiment with 46 lineages subjected to regular single colony bottlenecks prior to clonal regrowth. Upon conclusion, we sequenced one clonal-isolate genome per lineage. Given collective evolution for 85,323 generations across all lines, we calculate a per base-pair mutation rate of ∼5.5 × 10^−10^ per site per generation, one of the highest mutation rates among free-living eubacteria. Given our estimate of *µ*, we derived *N_e_* at ∼10^7^ from neutral diversity at four-fold degenerate sites across two dozen *M. xanthus* natural isolates. This estimate is below average for eubacteria and strengthens an already clear negative correlation between *µ* and *N_e_* in prokaryotes. The higher and lower than average mutation rate and *N_e_* for *M. xanthus*, respectively, amplify the question of whether any features of its multicellular life cycle—such as group-size reduction during fruiting-body development—or its highly structured spatial distribution have significantly influenced how these parameters have evolved.

**Significance**

Accurate estimation of mutation rates (*μ*) in different species is central to a mechanistic understanding of evolutionary processes and enables accurate inference of other fundamental population-genetic parameters from whole genome sequences (WGS), including effective population size (*N_e_*). Here we report the direct inference of *μ* and *N_e_* from WGS for *Myxococcus xanthus*, an important bacterial model for multicellular evolution and cooperation. This work is the first contribution to filling an important gap in our knowledge of mutation rates of multicellular prokaryotes.

## Introduction

*De novo* mutation is the ultimate source of genetic variation that fuels evolutionary change. The rate at which mutations occur spontaneously is a fundamental parameter for understanding evolutionary processes (Lynch et al. 2016). Prior to the next-generation sequencing (NGS) revolution, intrinsic mutation rates in bacteria were estimated primarily by either Luria-Delbrück fluctuation tests (Luria and Delbrück 1943) under selective experimental conditions (Drake 1991; Lynch 2010a) or by comparing sequences of lineages with independently estimated divergence times (Ochman et al. 1999). Both approaches have limitations, and mutation-rate estimates from the different methods for the same organism can vary by orders of magnitude (Drake 1991; Ochman et al. 1999). With the advent of NGS, direct mutation-rate estimation from whole genome sequences (WGS) became the gold standard (Lynch et al. 2016). In this approach, single clonal isolates are sampled and sequenced from controlled long-term evolution experiments and rates are inferred from accumulated mutations across many replicate lines, with mutation rates in *Escherichia coli* being among the first to be estimated directly in this way (Wielgoss et al. 2011; Lee et al. 2012b). To minimize possible effects of selection, mutation-rate estimates in clonally reproducing organisms are now commonly inferred from mutation accumulation (MA) experiments in which evolving populations are repeatedly reduced to a single cell on fresh solid-culture medium, typically by restreaking. The frequent introduction of single colony bottlenecks persistently imposes low effective population sizes (*N_e_*) at which genetic drift dominates evolution and mutations largely accumulate following the underlying spontaneous mutation rate as long as the selection coefficient *s* < |1/*N_e_*| (Lynch 2008; Halligan and Keightley 2009; Barrick and Lenski 2013; Lynch et al. 2016).

Importantly, precise laboratory estimates of mutation rates can be combined with estimates of neutral sequence diversity in natural populations to arrive at an estimate of another fundamental evolutionary parameter: the effective population size. Effective population size determines the strength of genetic drift in a population. Mathematically, the effective population size is conceived as the size of an ideal population that undergoes no selection but, evolving at a defined mutation rate, produces the same amount of neutral genetic diversity as observed in the real population (Fraser et al. 2009; Wielgoss 2022). Consequently, it has important implications for the long-term survival and adaptability of populations and is predicted to be the most important determinant of mutation-rate levels, according to the drift-barrier hypothesis (Lynch et al. 2016).

One major form of evolutionary innovation—obligate multicellularity with a single-cell life-history stage—is associated with lowered effective population sizes, and correspondingly, higher mutation rates, relative to both eukaryotes and prokaryotes considered to be unicellular (Lynch et al. 2023). However, other forms of multicellularity have not been similarly well examined for possible effects on mutation-rate evolution. While mutation rates of over two dozen eubacterial and several archaebacterial species have been estimated using MA experiments and *N_e_* has been estimated for most of these species as well (Bobay and Ochman 2018; Chen et al. 2022; Pan et al. 2022), they do not include species that exhibit complex forms of prokaryotic multicellularity such as aggregative fruiting-body formation (La Fortezza, Schaal et al. 2022) or filamentous division of labor (van Gestel et al. 2015). Here we begin to fill this gap by reporting two key population-genetic parameters, mutation rate (*µ*) and effective population size (*N_e_*), for the fruiting-body-forming bacterial predator *Myxococcus xanthus*. Mutation rates were inferred from a long-term MA experiment with 46 independent lineages, and *N_e_* was estimated from neutral genetic variation at four-fold degenerate sites in aligned single-copy genes of two dozen natural isolates.

*Myxococcus xanthus* belongs to the phylum *Myxococcota* (Waite et al. 2020; Parks et al. 2022) and is a model for studying microbial multicellular development, social evolution, and predator–prey interactions (Velicer and Vos 2009). Exposed to starvation stress, groups of otherwise free-living *M. xanthus* cells aggregate to form multicellular fruiting bodies wherein a minority of cells differentiate into stress-resistant spores that can survive long periods of nutrient deprivation, while the remaining majority of non-spore cells are likely to have lower prospects for survival under prolonged starvation. Many aspects of this highly cooperative organism's multicellular life cycle have been studied in great molecular detail (Mauriello et al. 2009; Yu et al. 2010; Nan et al. 2013; Rajagopalan et al. 2015; Faure et al. 2016; Gallego-Garcia et al. 2019; Vassallo and Wall 2019; Treuner-Lange et al. 2020; Islam et al. 2023).

The relative ease of handling and culturing *M. xanthus* facilitates the use of this species for evolution experiments. Evolution experiments with myxobacteria, or “MyxoEEs” (myxoee.org)(Rendueles and Velicer 2020), have enhanced our understanding of the evolution of microbial sociality (Velicer and Vos 2009), including social development (Velicer et al. 2000; Yu et al. 2010; La Fortezza and Velicer 2021), social motility (MyxoEE-3: Velicer and Yu 2003), and predator–prey interactions (MyxoEE-3: La Fortezza, Rendueles, et al. 2022; MyxoEE-4: Hillesland et al. 2009; MyxoEE-6: Nair et al. 2019). Moreover, several aspects of *M. xanthus* biogeography in nature have been characterized. These include the demonstration of isolation-by-distance across natural populations represented by soil isolates sampled from local to global scales (Vos and Velicer 2008; Kraemer et al. 2016), the genomic composition of multicellular fruiting bodies isolated from soil (Kraemer and Velicer 2011; Wielgoss et al. 2019), the character of social interactions between genotypes derived from the same or from different natural fruiting-body groups (Fiegna and Velicer 2005; Vos and Velicer 2009; Rendueles et al. 2015; Kraemer et al. 2016; Pande and Velicer 2018), and relevant evolutionary drivers of this diversity (Kraemer et al. 2016; Wielgoss et al. 2016; Wielgoss et al. 2019). Knowledge of the intrinsic *M. xanthus* mutation rate is crucial for better understanding evolutionary rates of this species in both natural and laboratory settings, as well as for testing for possible effects of prokaryotic multicellularity on mutation-rate evolution.

## Results

### Description of Mutations and Mutational Spectra

Our MA experiment—here named “MyxoEE-8” —was conducted on a nutrient-rich medium under non-stressing laboratory conditions with parallel replicate lines of a non-clumping strain of *M. xanthus* (see Materials and Methods). The experiment lasted for 396 days and comprised 75 single colony bottlenecks. We obtained *Illumina* paired-end sequences for 46 final time-point isolates that had each undergone an average of ∼1,855 generations of binary fission for a total of ∼85,323 generations across all lines. We estimated the average experimental effective population size due to repeated single colony bottlenecks to be low at ∼25 per lineage.

**Fig. 1.**
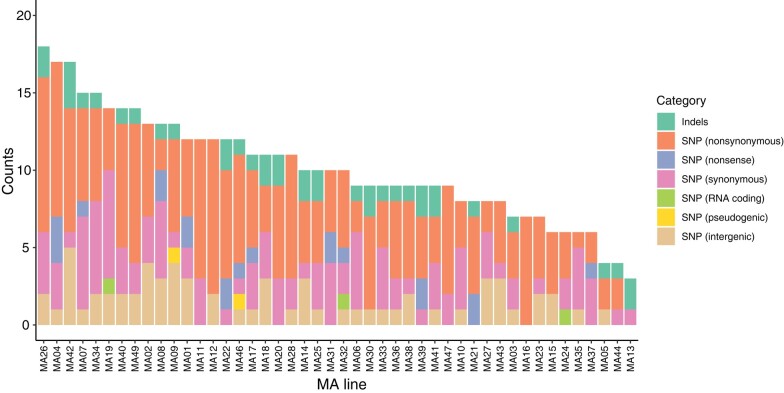
Plot of total number of mutations per MA line.

After detecting and removing 11 universally shared differences relative to the published sequence of strain DK1622 (supplementary table S1, Supplementary Material online), including all previously detected mutations (Velicer et al. 2006), we identified a total of 428 *de novo* base-pair substitutions (referred to as single nucleotide variants, SNVs) and 22 insertions and 17 deletions (Indels, ranging from 1–16 bp and 1–5 bp, respectively) across all sequenced lineages with high confidence (Fig. 1 and supplementary tables S2 and S3, Supplementary Material online). We did not detect any movement of insertion sequences (IS) or larger structural changes. Each genome was composed of ∼9.08 Mbp uniquely mapped positions with an average coverage ∼138 per site. Hence, ∼99% of the ancestor's ∼9.14 Mbp-genome size was adequately covered. Mutations were Poisson-distributed across lineages (1-sample Kolmogorov–Smirnoff test: *P* = 0.37; *D* = 0.13) with an average of 10.2 fixed mutations per MA line. Among SNVs, 358 (∼83.6%) were found in protein-coding regions, which represent ∼90.3% of the ancestral genome sequence, while the remaining 70 were found in intergenic and non-protein-coding sites (65, intergenic; 2, pseudogenic; 3, RNA-coding; supplementary table S3, Supplementary Material online). One of the coding SNVs has amino acid code-changing effects on two overlapping open reading frames (G → A change at genomic position 6,896,187 in line MA39). We counted this event as a single nonsynonymous SNV.

### Mutation-Rate Estimate

We calculated the maximum likelihood values of the various per lineage base-pair mutation rates from a Poisson distribution conditional on the mutational targets (Table 1). From this, the overall *M. xanthus* rate *µ* is estimated to be 5.5 (Confidence interval (CI 95%): 5.0–6.1) × 10^−10^ per site per generation, or 0.0050 (CI 95%: 0.0046–0.0055) mutations per genome per generation. We also infer the joint Indel mutation rate at 5.0 (CI 95%: 3.6–6.9) × 10^−11^ per site per generation, an order of magnitude lower than the base-pair substitution rate. Among all Indels (Table 1 and supplementary table S5, Supplementary Material online), ~69% (27/39) were additions or reductions of single units in simple tandem repeats (STR), also referred to as microsatellites (Goldstein and Schlötterer 1999). Microsatellites display highly elevated mutation rates mainly due to uncorrected strand-slippage of the polymerase during DNA replication in various organisms, including in bacteria (Levinson and Gutman 1987). We confirm this expectation and find that STR-type mutations occur at a per-target-site rate up to ∼820-fold higher than that of non-STR mutations (Table 1).

**Table 1:** Summary table of mutation-rate estimates for different genomic targets and mutational classes

Target	Target size	Mutations	*µ* (per site per generation)^a^ × 10^-10^	*µ* (per genomic target per generation)^a^
SNV, Total	9,080,403	428	5.52 (5.01–6.07)	0.0050 (0.0046–0.0055)
SNV, Nonsynonymous	6,053,873	249	4.82 (4.24–5.46)	0.0029 (0.0026–0.0033)
SNV, Synonymous	2,143,616	109	5.96 (4.89–7.19)	0.0013 (0.0010–0.0015)
SNV, Intergenic	828,655	65	9.19 (7.10–11.7)	0.0008 (0.0006–0.0010)
SNV, Pseudogenic	47,343	2	4.95 (0.60–17.9)	0.00002 (0.000003–0.00008)
SNV, RNA-coding	6,917	3	50.8 (10.5–149)	0.00004 (0.000007–0.00010)
Indels, Total [Insertions/deletions]	9,080,403	39 [22/17]	0.50 (0.36–0.69)	0.00046 (0.00033–0.00062)
Indels, non-STR	9,080,403^b^	12 [1/11]^c^	0.15 (0.08–0.27)	0.00014 (0.00007–0.00025)
Indels, STR^d^	25,664^e,f^	27 [21/6]	123 (81.3–179)	0.00032 (0.00021–0.00046)

^a^Numbers in brackets signify 95% confidence intervals.

^b^Non-STR mutations can occur at all covered sites.

^c^Exclusively found in coding regions.

^d^STR, simple tandem repeats.

^e^STR mutations only happened at certain focal STR loci, here, tandemly repeated mono- (A, T, G, or C), tri- (ATG), penta- (GCCGG) or hexadeka- (CAGAGCCTCGAAATCA)-nucleotide motifs.

^f^Converted to number of STR units (equals 31,488 bp) to correct for the fact that single mutational events are all single unit additions or reductions of whole motifs rather than single base-pairs, in general.

### Lack of Selection and Presence of Mutational Biases

We now present tests that seek to detect biases in the mutation types present in evolved clones to (i) detect any evidence of selection having operated significantly in our experiment and, in the absence of such evidence, (ii) potentially detect any biases in the types of mutations that occurred in our lineages. First, we calculate the ratio of nonsynonymous or nonsense (249) versus synonymous (109) SNV mutations at ∼2.3, which does not differ significantly from an expected value of ∼2.8 calculated from the potential targets for generating each mutation type (supplementary table S4, Supplementary Material online), i.e. 6,053,873 bp (nonsynonymous) versus 2,143,616 bp (synonymous; *χ*^2^ = 3.2, df = 1, *P* = 0.073). Hence, there is no clear evidence of selection having operated significantly in our MA experiment based on single nucleotide replacements.

**Fig. 2.**
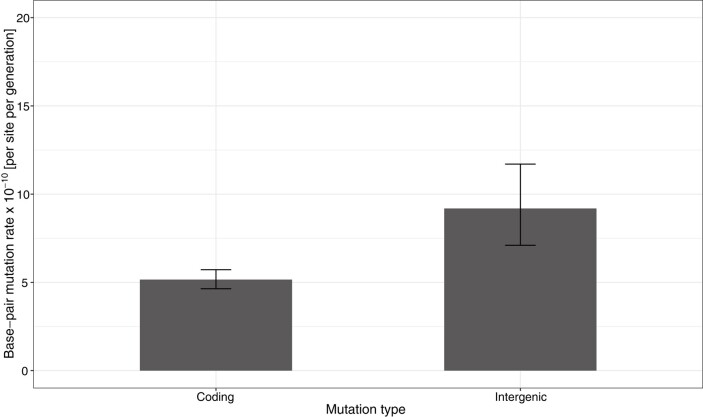
Base-substitution mutation rates in coding and intergenic regions. Error bars depict 95% confidence intervals.

**Fig. 3.**
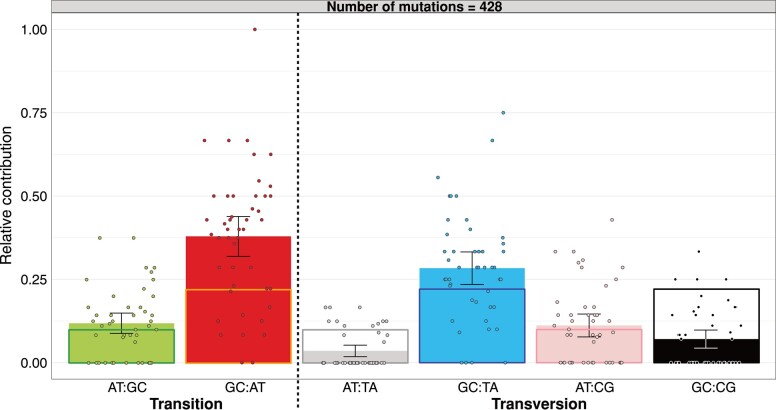
Observed and expected mutational spectra for point mutations. Points depict mutational data per MA line, bars depict average values per category across MA lines, and error bars depict 95% confidence intervals (each category is Poisson-distributed). Expected mutations were calculated from the average number of available sites at risk to produce a mutation in a category (bold rectangle outlines around each barplot).

**Fig. 4.**
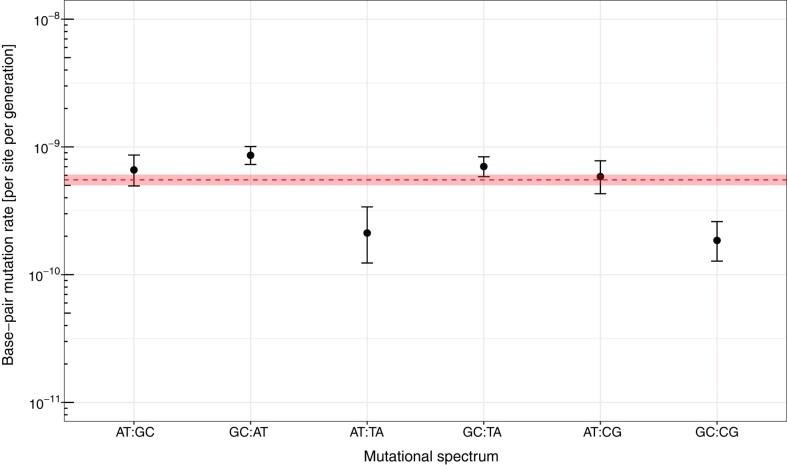
Conditional base-substitution mutation rates. The dotted line indicates the overall base-substitution mutation rate across all spectra and the shaded area represents the respective 95% confidence interval. Error bars depict 95% confidence intervals per each mutational category; all categories are Poisson-distributed.

In contrast, we did detect a very large and significant bias in the distribution of mutations present in coding versus non-coding regions relative to the expectation under randomness. Of the 428 total SNVs, there are significantly fewer base-pair changes in the total number of protein- (358) and RNA-coding genes (3) than in the joint number of intergenic (65) and pseudogenic regions (2) relative to expectations from the total (translated and untranslated) coding (∼90.4%) versus inter- and pseudogenic (∼9.6%) fractions of the genome (*χ*^2^ = 17.0, df = 1, *P* = 3.67 × 10^−5^). This bias is driven by a mutation rate of 9.19 (CI 95%: 7.10–11.7) × 10^−10^ per site per generation in the intergenic region, which is ∼1.8-fold higher than the rate in protein-coding portions of the genome of 5.16 (CI 95%: 4.64–5.72) × 10^−10^ per site per generation (Fig. 2). We also tested if the Indel distribution deviated from random expectations. Out of 39 insertions and deletions, 25 were found in coding and 14 in intergenic regions, which deviates similarly strongly from random expectations of 35 versus 4 (*χ*^2^ = 27.9, df = 1, *P* = 1.28 × 10^−7^). However, the nature of the distribution is likely driven by mutational bias, i.e. strand-slippage-inducing microsatellite loci across the entire genome, rather than selection. We come to this conclusion because the majority of Indels (27/39) are mutations in the hyper-mutable STR category, and the remaining Indels (12) are exclusively situated in protein-coding genes (Table 1), the latter of which would be expected by chance (*χ*^2^ = 0.41, df = 1, *P* = 0.52). Beside an enrichment of mutations in non-coding regions, we also observe strong evidence for mutation-type SNV biases in our data. First, we conducted a dispersion test to confirm that the mutational count data within each mutational base-pair-change category adheres to the assumptions of a Poisson distribution (supplementary table S6, Supplementary Material online). We then confirmed that there are significantly fewer AT:TA and GC:CG changes and more GC:AT and GC:TA changes than expected by chance (Figs. 3 and 4). Additionally, the observed ratio of transitions versus transitions and transversions (206 and 222, respectively) at ∼0.48 deviates significantly from the null expectation of 1/3 under a uniform mutation rate (exact binomial test, *P* ∼ 2.9 × 10^−10^). Moreover, among all base-pair changes, changes toward A or T (295) outnumber changes toward G or C (133), so that the relative ratio of observed changes toward A or T (∼0.69) deviates significantly from the null expectation of ∼0.56 assuming a uniform mutation rate and accounting for the genome's GC content of ∼69% (exact binomial test, *P* ∼ 9.6 × 10^−8^).

### Estimation of Effective Population Size From Natural Isolates

**Fig. 5.**
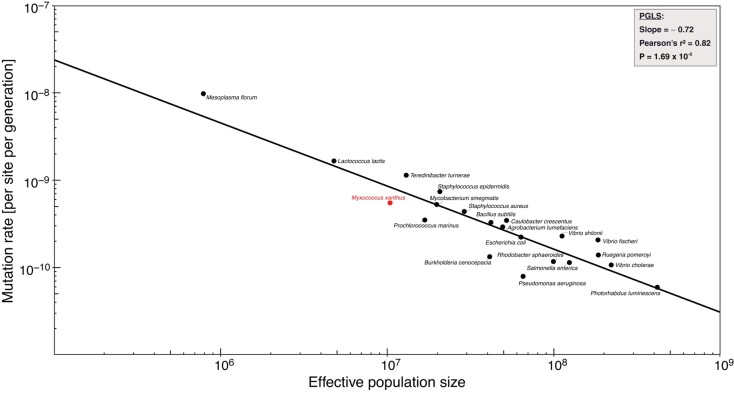
Correlation of effective population size (*N_e_*) and base-pair mutation rate (*µ*) for different bacterial species (*n* = 21).

Finally, after accounting for population structure, we estimated the natural effective population size *N_e_* for *M. xanthus* (assuming haploidy) from *π* = 2*N_e_µ*, where the parameter *π*, nucleotide diversity, was measured at four-fold degenerate sites in conserved single-copy genes across 24 natural isolates (supplementary fig. S1, Supplementary Material online). Given *π* = 0.011 and using our reported per base-pair mutation rate of *µ* = 5.52 × 10^−10^ per site per generation, we derive an *N_e_* value of ∼10^7^. To put this estimate into perspective, we retrieved and plotted mutation-rate values (*µ*) over *N_e_* estimates for *M. xanthus* and additional eubacterial species with data derived using an equivalent estimation approach (Chen et al. 2022), replacing their estimates for *Salmonella enterica* with the more recently and precisely calculated values of *µ* and *N_e_*(Pan et al. 2022). Applying a linear model based on phylogenetic least squares (PGLS), we find a close and strongly negative correlation between *µ* and *N_e_* (*n* = 21; slope = −0.72; Pearson's *r*^2^ = 0.82; *P* = 1.69 × 10^−8^; Fig. 5). We performed the same analysis for *µ* over genome sizes, for the same data points, and found that both parameters also scaled strongly negatively, albeit with a much lower correlation coefficient (*n* = 21; slope −0.95; Pearson's *r*^2^ ∼ 0.19; *P* = 0.047; supplementary fig. S2, Supplementary Material online).

## Discussion

Here we present two of the most fundamental population-genetic parameters, *µ* and *N_e_*, for the model bacterium *M. xanthus* with unprecedented accuracy. Our analyses indicate that *M. xanthus* has one of the highest mutation rates among free-living eubacteria (Fig. 5). Additionally, in line with the general trend across bacteria (Sung et al. 2016), the Indel mutation rate in *M. xanthus* is ~10-fold reduced relative to the base-pair mutation rate, and is mainly driven by mutations in microsatellites.

Multiple biases in the character of SNVs shape the spectrum of *de novo* mutation in *M. xanthus*. First, we found a clear transition bias in new mutations of ∼1.4-fold, an estimate that agrees well with previous MA mutation-rate studies in bacteria (Katju and Bergthorsson 2019) and appears to be a systematic bias across organismal systems (Stoltzfus and McCandlish 2017). Second, we also found a strong bias of mutations occurring from G or C toward A or T compared to vice versa. It was long thought that genomic enrichments of G and C or A and T have arisen via highly skewed mutational processes (Sueoka 1962; Muto and Osawa 1987). However, while *M. xanthus* has highly elevated GC content of ∼69%, similar to many other soil bacteria (Foerstner et al. 2005), only ∼31% of new mutations are toward G or C from A or T, a bias opposite in direction from the high GC content of the genome. Based on previous independent observations, a bias of excess mutations from G or C toward A or T appears likely to be common among microbes, irrespective of GC-composition (Hershberg and Petrov 2010; Hildebrand et al. 2010). Thus, GC-enrichment of genomes is generated by processes other than mutation bias, likely a combination of selective and neutral processes (Hershberg 2015). Among non-selective processes, biased gene conversion (see Duret and Galtier 2009 for a review), and efficacy and functionality of mismatch repair systems (Lee et al. 2012b) have been implicated to impact genomic GC content in bacteria. In contrast, a recent study with multiple strains of *E. coli* indicates that the evolutionary history of a species could play a crucial role in shaping skewed mutational spectra, and that evolutionary shifts in genomic bias do not necessarily rely on specific underlying mechanisms or selection favoring particular types of mutations (Sane et al. 2023).

Hypothetically, the significantly lower number of base-pair changes in coding regions than expected by chance might have occurred either because (i) selection strongly impacted the outcome of our MA experiment and disproportionately acted against coding-region mutations or (ii) the actual mutation rates of coding versus non-coding regions differ. Regarding the first hypothesis, multiple considerations suggest that only a small minority of mutations in our MA experiment were influenced by selection. If the selection hypothesis were correct, the bias against coding-region mutations should have been accompanied by a significant bias against nonsynonymous mutations relative to synonymous SNV mutations within coding regions, understanding that some synonymous mutations can affect fitness (Lebeuf-Taylor et al. 2019). But this was not the case, suggesting that coding-region mutations accumulated in a predominantly neutral manner. In fact, synonymous SNVs exceed the number expected by chance by a factor of only ∼1.2. Additionally, with an average experimental *N_e_* of ∼25, the magnitude of the threshold selection-coefficient (*s*) required to confer a deleterious or adaptive effect in our settings would need to exceed |1/*N_e_*|, or ∼0.04, and historically only a small minority of new mutations have been understood to have such large fitness effects (Kibota and Lynch 1996); see also Sane et al. (2023). We thus infer that the 1.8-fold excess of mutations in non-coding regions relative to the expectation under randomness is predominantly due to a difference in the rate at which unrepaired mutations are generated in the two types of genomic region.

The combination of elevated MA in intergenic regions without a corresponding bias in nonsynonymous versus synonymous mutation rates within coding regions has been reported for several previous MA experiments with bacteria, including for *Burkholderia cenocepacia* (Dillon et al. 2015), *E. coli* (Lee et al. 2012b), and two *Vibrio* species (Dillon et al. 2017). The contrasting trends have been hypothesized to result from preferential mutation-mismatch repair of damage in coding regions in bacteria, thereby introducing a molecular signal that may mimic signals of selection on non-coding regions (Lee et al. 2012b). It also appears reasonable that evolutionary changes in mutation-repair genes may explain variation in mutational skews across bacterial groups (Sane et al. 2023). Finally, the observed pattern in bacteria is somewhat reminiscent of mutation distributions in *Arabidopsis thaliana* observed after MA experiments by Monroe et al. (2022). The latter study suggests that mutational processes are strongly biased toward higher mutation rates immediately outside of gene bodies due to protective masking via epigenetic markers, as there was no evidence of selection within coding portions. Further research is needed to identify the molecular mechanism underlying the observed bias in *M. xanthus* and other bacteria.

It is intriguing that the mutation rate for *M. xanthus* is among the highest rate estimates in bacteria, with the per-genome rate (0.0046) exceeding that of the model organism *E. coli* by >10-fold (Wielgoss et al. 2011; Lee et al. 2012b). This high mutation rate for *M. xanthus* illustrates that some early predictions for mutation-rate evolution in microbial genomes do not hold for eubacteria in general. These predictions include that (i) per-nucleotide-per-generation mutation rates would generally negatively scale with genome size—the *M. xanthus* genome is much larger than average—and (ii) there is a universal per-genome-per-generation mutation rate of ∼0.003 (Drake 1991). While how genome size might affect point mutation rates across the microbial realm remains unclear, the drift-barrier hypothesis for mutation-rate evolution has been proposed to explain the negative relationship between mutation rate and effective population size observed across species (Lynch et al. 2016, 2023). In line with the drift-barrier hypothesis, and assuming that most natural *M. xanthus* lineages have mutation rates similar to that of the lab strain we examined, we estimate an effective population size of ∼10^7^ for natural *M. xanthus* populations. This estimate is below the average of *N_e_* values calculated for all 21 included bacterial species (Chen et al. 2022; Pan et al. 2022) and strengthens the already clear negative correlation between *µ* and *N_e_* among eubacteria.

Several aspects of natural populations can impact *N_e_* (Caballero 1994), some of which are specific to sexual organisms (e.g. sex ratio) and some of which are universal, such as spatial dispersion (Woolfenden and Fitzpatrick 1984) and population size fluctuation over time (Vucetich et al. 1997), with highly viscous and highly fluctuating populations having smaller effective populations sizes relative to their observed census sizes. Both of the latter population features may contribute to the lower-than-average *N_e_* of *M. xanthus*. Given its wide geographic distribution in top-soils of the world (Dawid 2000), *M. xanthus* is likely to have a large global average census population size. However, *M. xanthus* resides extensively in highly structured soil environments, and natural genetic diversity in this species is similarly highly spatially structured. Populations show evidence of isolation-by-distance not only at global scales but also at fine local scales (Vos and Velicer 2008; Kraemer et al. 2016), consistent with the possibility that *M. xanthus* populations may be more spatially viscous than those of many other bacterial species. It is also possible that local *M. xanthus* populations fluctuate in size over time more than do those of many other species. Many cells entering fruiting-body development are thought to die in the process, and only a minority of cells entering development become stress-resistant spores, at least under laboratory conditions (Wireman and Dworkin 1977; O’Connor and Zusman 1988; Lee et al. 2012a), suggesting that starvation-induced multicellular development may often be associated with large local population reductions. However, the population dynamics of development have not been studied under natural conditions. Additionally, recent research on genomic diversity within single fruiting bodies from soil (Wielgoss et al. 2019) has shown that, while fruiting-body groups examined at one point in time generally reflect internally diverse clusters of highly related lineages that are predicted to stay spatially associated for hundreds of generations, it is also predicted that single-cell bottlenecks nonetheless considerably reduce within-group genetic variation frequently. Further research comparing degrees of population viscosity and population size fluctuation across bacterial species in their natural habitats will be required to assess the relative degrees to which these population features can explain the substantial variation in effective population sizes exhibited by bacteria.

The emergence of gametic multicellularity clearly had important consequences for the evolution of effective population size and mutation rate, substantially lowering and raising these parameters, respectively, on average (Lynch 2008, 2010a, 2010b). Our results offer initial data points needed to ultimately assess whether some forms of prokaryotic multicellularity may also influence the evolution of these parameters. Several bacterial species not known to exhibit complex aggregative multicellularity have higher mutation rates than *M. xanthus* (Fig. 5) and factors other than multicellularity can shape *N_e_* and mutation rates (Lynch et al. 2023). Nonetheless, local population fluctuations associated with developmental cell death as well as features of *M. xanthus* demographic dynamics may have contributed to the evolution of its relatively high mutation rate. Intriguingly, *Dictyostelium discoideum*—a eukaryote exhibiting a process of aggregative fruiting-body development analogous to that of *M. xanthus* (La Fortezza, Schaal, et al. 2022)—has one of the lowest mutation rates among eukaryotes (Kucukyildirim et al. 2020). However, in contrast to the consensus view of *M. xanthus*, a large majority of *D. discoideum* cells survive the developmental process (Kessin 2001). Clearly, much more work is needed to understand whether and how different forms of microbial multicellularity influence mutation-rate evolution.

As a final note, it appears that not all myxobacteria form fruiting bodies (Jiang et al. 2007). Hence, one possible approach for this clade then, among others, would be to estimate mutation rates for numerous myxobacterial species that vary in their ability to form fruiting bodies, as well as for several non-myxobacteria species that are phylogenetically proximate to the myxobacteria. This would allow analysis of whether mutation rates appear to vary in a manner associated with the presence of aggregative development or not.

## Materials and Methods

### MA Experiment

A single clone of ancestral strain GJV71 was used to set up the independent MA lineages. GJV71 is a Δ*cglB* Δ*pilA* double deletion mutant of strain GJV1, a derivative of reference strain DK1622 (Velicer et al. 2006). GJV71 is defective at both forms of *M. xanthus* motility, namely social (S) motility (Chang et al. 2016; Treuner-Lange et al. 2020) and adventurous (A) motility (Nan et al. 2013; Faure et al. 2016). The double deletion ensured that cell divisions and not active movement drive colony growth. Lineages generally went through 75 single colony bottlenecks of picking and restreaking on Casitone-Tris (CTT) (Bretscher and Kaiser 1978) complex medium supplemented with 1.5%-agar, with a median of 5 d between transfers. At regular intervals throughout the experiment, glycerol (10%, v/v) stocks of each line were prepared and stored at −80 °C until further processed. We assessed the average number of generations between transfer bottlenecks as follows. Overnight cultures were grown in liquid CTT medium, diluted, and plated on CTT (1.5%) agar plates. Six independent (single) colonies were picked and resuspended (and this for three different time-points, i.e. after 4, 5, or 6 d of growth). Each resuspension was diluted and plated on CTT (1.5%) agar plates, to assess average cell numbers of single colonies, and hence doubling times after 4, 5, or 6 d prior to transfer. From this we calculated the average number of cell divisions per day (∼4.8), per transfer (∼25, i.e. after ∼5.2 d) and throughout the entire MA experiment (at ∼1,855 generations per lineage after 75 transfers; supplementary table S3, Supplementary Material online). As a remark, while most lineages had the maximal number of generations, some lineages needed to be restarted from freezer stock of a previous transfer at some point during the experiment. This was because a lineage either did not show growth at a particular time-point or because there was a non-myxobacterial contaminant on the agar. This procedure thus slightly reduced the overall number of possible generations (supplementary table S3, Supplementary Material online). Finally, we can estimate the experimental effective population size from the number of generations (cell divisions) per transfer, *g*, and the bottleneck size, *N_b_*, from *N_e_* = *g* × *N_b_* (Wielgoss 2022), which gives the same result as the harmonic mean. Assuming that each colony originates from a single cell (*N_b_* = 1), it follows that the average *N_e_* across lineages is equal to the average number of generations per transfer (∼25).

### Whole Genome Resequencing

A total of 46 lineages were revived from glycerol stocks of the last time-point on CTT solid medium. After around 5 d, a single colony (representing transfer 75) was directly picked into 8 mL of CTT liquid medium (same recipe as above, without agar) and incubated for another 2–3 d until surpassing exponential phase (OD_595nm_ > 0.5). Cells were pelleted by centrifugation (15 min at 5,000*g*), and stored at −80 °C until further processing. DNA extraction was performed using Qiagen's genomic DNA extraction kit and using 20/G genomic tips according to the manufacturer's recommendations. DNA was diluted in 10 mM Tris (pH = 8.0) and directly shipped to two different sequencing facilities (Quantitative Genomics Facility, D-BSSE, ETH Basel, Switzerland, and FASTERIS, Geneva, Switzerland). At ETH Basel, libraries were prepared using the NEBNext DNA Kit with average insert-sizes of 600 bp. At FASTERIS, libraries were prepared using the Nextera XT DNA Kit with average insert-sizes of less than 300 bp. At both sequencing facilities, sequences were pooled on one lane of an Illumina HiSeq2500 machine to yield 125 bp sequences in paired end mode with an average coverage of ∼137 per genome.

### Resequencing Analyses

Paired reads were very stringently cleaned from low-quality and adapter sequences using *Trimmomatic* v0.32 (Bolger et al. 2014) with the following specific parameters for paired-end read data:

*ILLUMINACLIP:Nextera+TruSeq3-PE2.fa:2:25:10 CROP:115 HEADCROP:10 LEADING:30 TRAILING:28 SLIDINGWINDOW:4:28 MINLEN:77*

The file “Nextera+TruSeq3-PE2.fa” combines two of trimmomatic's provided adapter files using the command-line command (inside trimmomatic's main folder):

*cat ./adapters/NexteraPE-PE.fa ./adapters/TruSeq3-PE.fa >* Nextera+TruSeq3-PE2.fa

Trimmed reads (paired and unpaired) were mapped against the closed reference genome of *M. xanthus* DK1622 (accession number: NC_008095.1; RefSeq version: 07-FEB-2021; Goldman et al. 2006) using *breseq* v0.35.5 (Barrick et al. 2014; Deatherage and Barrick 2014) with the following general parameters:

*breseq -j 2 --minimum-mapping-quality 25 -r* NC_008095. gbk *fastq.gz

The *breseq* pipeline depends on several programs including *Bowtie2* v2.3.2 (Langmead and Salzberg 2012) and *R* (v4.2.3; “Shortstop Beagle”; R Core Team 2023).

The mapping output in gd-file format was analyzed using breseq's *gdtools* commands:

(a) for annotation: *gdtools ANNOTATE*
(b) for removing shared mutations: *gdtools SUBTRACT*
(c) for basic mapping statistics: *gdtools COUNT -b*
(d) for conversion to Variant Call Format (VCF): *gdtools GD2VCF*

Prior to downstream analyses, we separated the 11 universally shared ancestral mutations relative to the reference genome of *M. xanthus* DK1622 (supplementary table S1, Supplementary Material online) using *breseq's* command *gdtools SUBTRACT*. The mutations represent the changes present in the experiment's ancestral clone. Among these differences were the five predicted mutational differences between the sequenced reference genome of strain DK1622 and our strain’s immediate ancestor GJV1 (Velicer et al. 2006), plus the two deliberately constructed deletions of 663 and 561 bp in genes *cglB* and *pilA*, respectively, plus a total of four additional mutations present in the starting isolate of GJV71 (supplementary table S1, Supplementary Material online). Mutations were inspected manually using binary alignment and map (BAM) files versus the reference with the Integrative Genomics Viewer (IGV) (Robinson et al. 2017), this encompasses mutations supported by >85% of mapping reads (mapping quality (MQ) > 25), with a mean read support of ∼99.6%.

Mutation-count data were derived from the *gdtools COUNT –b* option (supplementary table S4, Supplementary Material online). All subsequent statistical analyses were performed in *R* (v4.2.3; “Shortstop Beagle”; R Core Team 2023) using *Rstudio* (v2022.07.2 Build 576).

### Mutation-Rate Estimation

We verified that our total mutation data across mutational categories were Poisson-distributed (“ppois”) based on 1-sample Kolmogorov–Smirnov tests (*R* base function ks.test(), considering the observed mean mutations across all lines at ∼10.2). We also verified that base-pair mutations within each of the six mutational categories conformed to the assumptions of Poisson-distributed data (and were not biased by zero-inflation). For this we performed a dispersion test, which relies on the assumption that the mean of Poisson-distributed data equals its variance. The ratio between variance and mean for a count sample of *n* (assuming Poisson-distributed data) is required to follow a Chi-square distribution with *n* − 1 degrees of freedom. Performing this test, we are unable to reject the null hypothesis of a Poisson distribution (all *P* > 0.05) for any of the six mutational categories (supplementary table S6, Supplementary Material online). Since the number of mutations is small relative to the number of total sites mapped (428 events on a target of size 9.08 Mbp), and assuming that the number of back mutations should be negligible, we thus calculated the maximum likelihood number of mutations and 95% confidence intervals (CI 95%) from a Poisson distribution (R base function *poisson.test*() = rate.mut) and inferred the respective rates for SNV and Indel mutations using the equation: *µ* = mut/*nT*, with *µ* being the mutation rate, mut being the number of mutations, *T* is the time in total number of generations, and *n* representing the average number of observed genomic sites at risk to mutate in a certain category across lines. Finally, to visualize mutational spectra, we downloaded the WGS of strain *M. xanthus* DK1622 from Genbank, and converted and stored the fasta-file as a BSGenome reference object in *R*. We read the VCF output for our 46 MA lines, and computed and plotted the observed mutational spectra using R package *MutationalPatterns* (Manders et al. 2022).

### Scan for STRs

Around two-thirds of all Indels were STR mutations, and the latter are known to have highly elevated mutations rates, including in bacteria (Levinson and Gutman 1987). Hence, to infer the mutation rate at relevant STR loci, we inferred their joint target size from all mutated, tandemly repeated mono- (A, T, G, or C), tri- (ATG), penta- (GCCGG), or hexadeka (CAGAGCCTCGAAATCA)-nucleotide motifs across the genome, by scanning the DK1622 reference genome with the exhaustive repeat finder *perf v.0.4.6* (Avvaru et al. 2018). We required minimum motif lengths for each category (mono = 6, tri = 4, penta = 2, and hexadeka = 1). For convenience, we report the total number of repeat units (25,664) instead of the bp-target size (31,448 sites), since all STR were additions or reductions of repeat units. This is indicated in Table 1.

### Phylogenetic Correction of Correlations Involving Mutation Rate, Population, and Genome Sizes

To study the relationship between mutation rate (*µ*), effective population size (*N_e_*), and genome size (*G*) across prokaryotic lineages, we retrieved a comprehensive dataset of 21 phylogenetically distinct species previously inferred by Chen et al. (2022) (and replacing *µ* and *N_e_* with more recently derived estimates for *S. enterica*; Pan et al. 2022). Since correlations between the above parameters can be skewed when not properly accounting for phylogenetic relationships (Chen et al. 2022), we added the new data for *M. xanthus* to the existing alignments of the former study, and re-inferred the phylogenetic tree in *IQ-TREE* (Nguyen et al. 2015) as described. That phylogenetic tree was used to assess the pairwise linear relationships between *μ*, *N_e_*, and *G* across the 21 species using the PGLS method, implemented in the *R* package *caper* v.1.0.3 (Orme et al. 2023).

### Estimation of Effective Population Size

We estimated nucleotide diversity at four-fold degenerate sites for *M. xanthus* genomes. In more detail, we retrieved the whole genomes of 46 *M. xanthus* isolates comprising natural soil isolates and the reference genome of DK1622 (Goldman et al. 2006; Rajagopalan et al. 2015; Wielgoss et al. 2016, 2019), and annotated and aligned a set of 1,000 universally shared single-copy orthologs using the codon tree method in Bacterial and Viral Bioinformatics Resource Center v.3.29.20 (Davis et al. 2020; Olson et al. 2023). To avoid that hidden population structure inflates our population size estimates (Chen et al. 2022), we estimated population structure for the 46 genomes with PopCOGenT (Arevalo et al. 2019), which defines populations as “recent gene flow units”. From this analysis, we inferred that around half of our initial dataset (*n* = 24; supplementary fig. S1, Supplementary Material online) formed a large unstructured subpopulation, amenable to infer *π* and hence *N_e_*, while the remaining set split up into more than 10 subpopulations (all with sample sizes *n* < 6) and was discarded from further downstream analyses. The 1,000 single-copy gene alignments retrieved for the subset of 24 genomes were concatenated, and four-fold degenerate sites were parsed with rphast::get4d.msa (Hubisz et al. 2011). Mind that, for this step, we used the latest version *rphast* v1.6.9 which needs to be run on an earlier version of *R* (v3.4.3). We calculated nucleotide diversity *π* with pegas::nuc.div (Paradis 2010) in the standard *R* version, from the aligned, and concatenated four-fold degenerate sites dataset. Finally, we derived effective population size *N_e_* for *M. xanthus* assuming haploidy from equation *π* = 2*N_e_µ*, with *π* being the nucleotide diversity at four-fold degenerate sites across natural isolates, and *µ* being the base-pair mutation rate of 5.52 × 10^−10^ per site per generation.

## Supplementary Material

evae066_Supplementary_Data

## Data Availability

All resequencing data generated from the MA experiment have been deposited at the National Center for Biotechnology Information (NCBI) Short-Read Archive (SRA) under Bioproject PRJNA1018729 and will be publicly accessible upon publication. All *R* scripts and accompanying raw data are available on figshare (doi:10.6084/m9.figshare.25075421).
