# Supplementary material for: Mutation Rate and Effective Population Size of the Model Cooperative Bacterium Myxococcus xanthus
Source: Genome Biol Evol. 2024 Mar 25;16(5):evae066. doi: 10.1093/gbe/evae066 (PMC11069108; doi:10.1093/gbe/evae066)
Supplement: evae066_Supplementary_Data [file evae066_supplementary_data.zip › SOM_WIELGOSS_GBE_2024.pdf]

Supplementary Material

Mutation rate and effective population size of the model cooperative bacterium *Myxococcus xanthus*

Sébastien Wielgoss[1]*, J. David Van Dyken[2,3], Gregory J. Velicer[1,2]*

[1] Institute for Integrative Biology, ETH Zürich, 8092 Zürich, Switzerland

[2] Department of Biology, Indiana University, Bloomington, USA

[3] Department of Biology, University of Miami, Coral Gables, FL, USA

* Correspondence to: sebastien.wielgoss@env.ethz.ch; gregory.velicer@env.ethz.ch

Supplemental Figures & Tables

Supplementary Figure 1: Phylogenetic relationship between 24 *Myxococcus xanthus* isolates rooted in a closely related outgroup used to infer nucleotide diversity and population size.

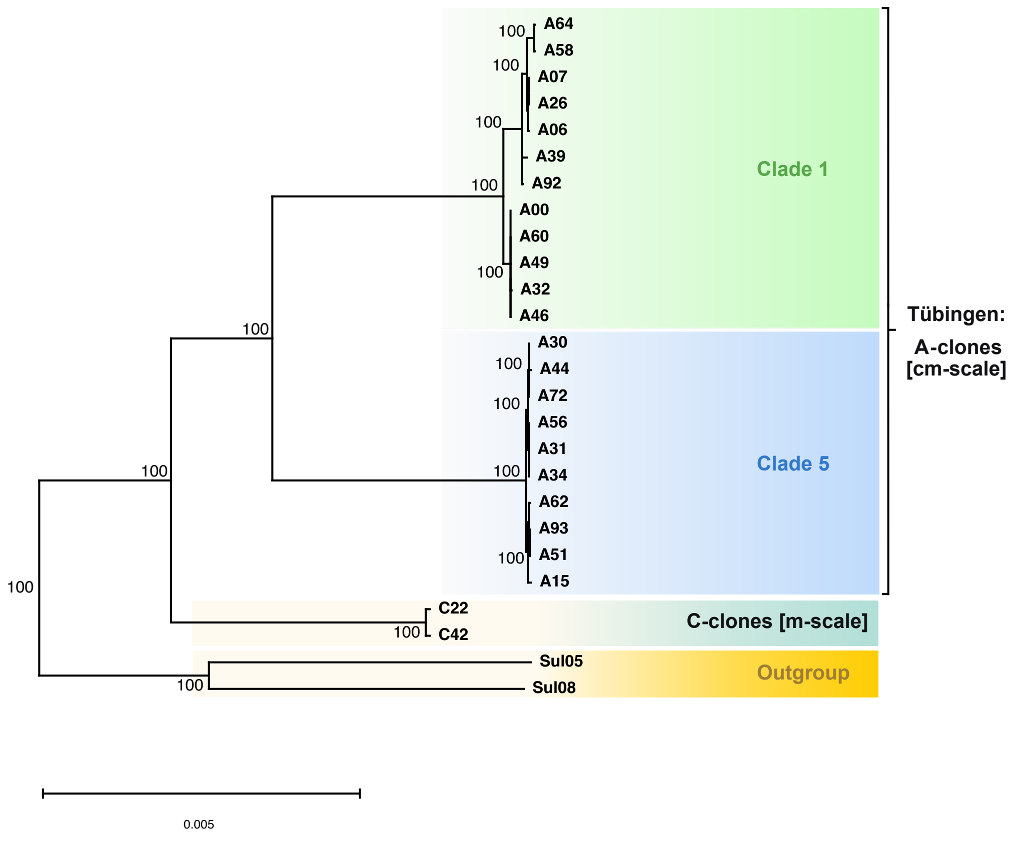

Supplementary Figure 2: Base-pair mutation rate and genome size are significantly negatively correlated for 21 eubacteria, including *M. xanthus*. Correlation was corrected for using phylogenetic relationship.

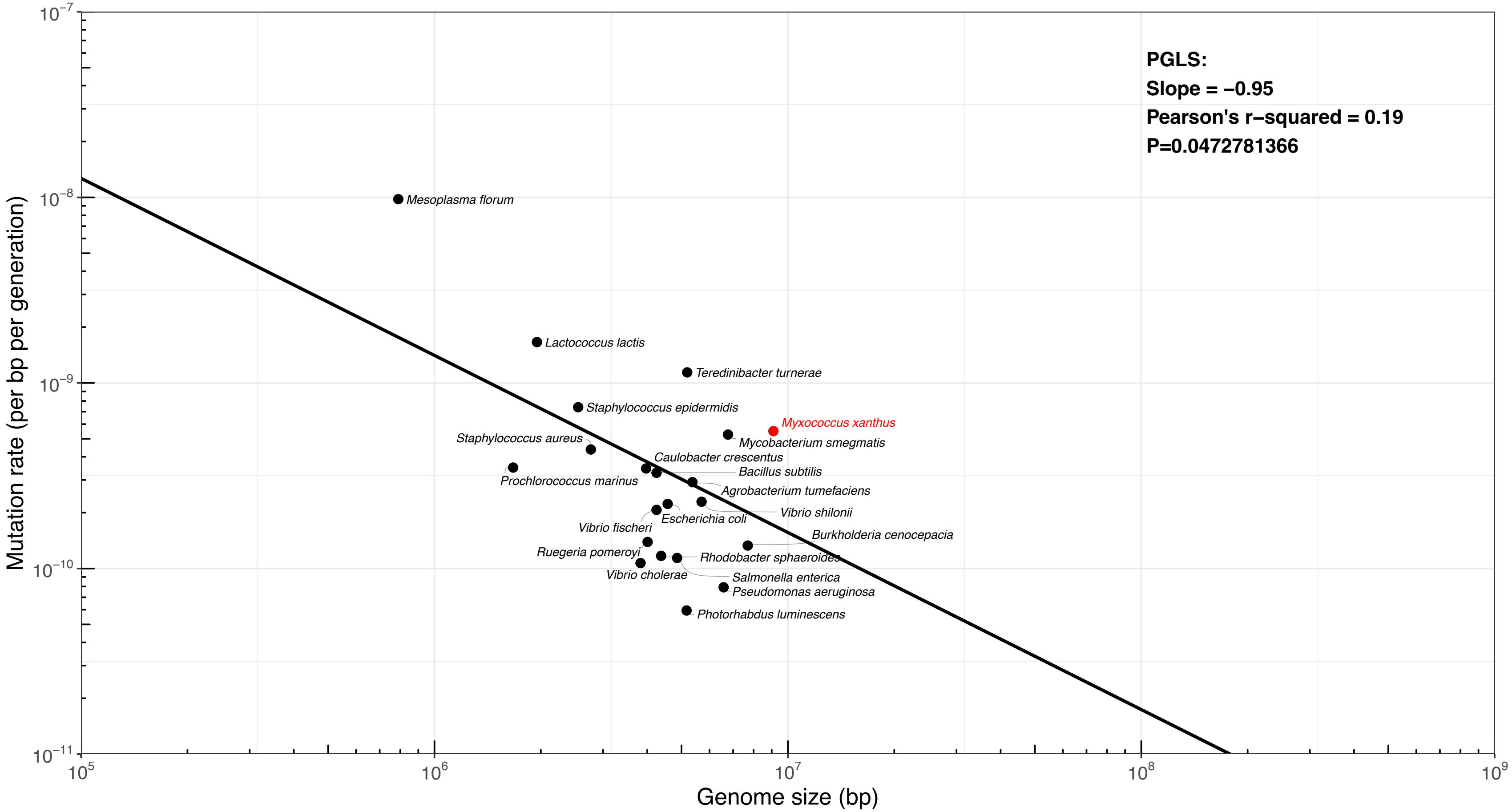

Supplementary Table 1: Universally shared and derived mutations across MA lineages relative to reference genome *M. xanthus* DK1622.

**Position**	**Category**	**Mutation**	**Annotation**	**Gene name**
830,180	SNV	T→G	intergenic	*MXAN_0726/MXAN_0729*
866,939	Insertion	$(G)_{7\rightarrow8}$	frameshift	*MXAN_0758*
1,717,943	SNV	G→T	synonymous	*MXAN_1458*
2,304,201	Deletion	Δ1 bp	frameshift	*MXAN_1970*
2,654,787	SNV	A→G	nonsynonymous	*dcp*
3,589,286	Deletion	Δ663 bp	large deletion	*cglB*
5,391,338	SNV	G→A	nonsynonymous	*kamD*
5,893,144	SNV	A→C	intergenic	*MXAN_4700/rrfC*
6,287,570	SNV	C→A	intergenic	*MXAN_5029/MXAN_5030*
7,101,832	SNV	T→C	synonymous	*ftsY*
7,158,853	Deletion	Δ561 bp	large deletion	*pilA*

SNV, single nucleotide variant.

Supplementary Table 2: Matrix detailing the nature, positions, and effects of all *de novo* mutations across MA lines relative to reference genome *M. xanthus* DK1622.

[Attached as separate original file: Wielgoss_GBE_2024_TableS2.xlsx]

Supplementary Table 3: Mutation summary

MA line	Time[1] [generations]	Mapped genome size[2] [bp]	SNV							Indel	
			non-synonymous	non-sense	synony-mous	non-coding	pseudo-genic	inter-genic	total	Insertions	Deletions
MA01	1866	9,084,224	5	2	2	0	0	3	12	0	0
MA02	1842	9,085,120	6	0	3	0	0	4	13	0	0
MA03	1866	9,084,862	3	0	2	0	0	1	6	0	1
MA04	1866	9,083,016	10	3	3	0	0	1	17	0	0
MA05	1842	9,085,528	2	0	0	0	0	1	3	1	0
MA06	1866	9,079,109	2	0	5	0	0	1	8	0	1
MA07	1842	9,082,293	6	1	6	0	0	1	14	1	0
MA08	1842	9,085,156	2	2	5	0	0	3	12	1	0
MA09	1866	9,073,837	6	0	1	0	1	4	12	0	1
MA10	1866	9,079,529	3	0	4	0	0	1	8	0	0
MA11	1842	9,085,098	9	0	3	0	0	0	12	0	0
MA12	1770	9,084,898	10	0	0	0	0	2	12	0	0
MA13	1866	9,084,861	0	0	1	0	0	0	1	1	1
MA14	1866	9,085,026	4	0	1	0	0	3	8	2	0
MA15	1866	9,085,002	4	0	0	0	0	2	6	0	0
MA16	1815	9,085,369	7	0	0	0	0	0	7	0	0
MA17	1866	9,085,097	5	1	3	0	0	1	10	0	1
MA18	1866	9,085,084	3	0	3	0	0	3	9	0	2
MA19	1866	9,085,001	4	0	7	1	0	2	14	0	0

MA20	1839	9,079,810	6	0	3	0	0	0	9	0	2
MA21	1866	9,085,127	5	2	0	0	0	0	7	1	0
MA22	1866	9,077,736	7	2	1	0	0	0	10	1	1
MA23	1866	9,076,231	4	0	1	0	0	2	7	0	0
MA24	1866	9,074,848	3	0	2	1	0	0	6	0	0
MA25	1866	9,076,923	4	0	3	0	0	1	8	1	1
MA26	1866	9,085,574	10	0	4	0	0	2	16	1	1
MA27	1842	9,076,981	2	0	3	0	0	3	8	0	0
MA28	1866	9,076,292	8	0	2	0	0	1	11	0	0
MA30	1842	9,076,892	6	0	0	0	0	1	7	2	0
MA31	1866	9,085,397	4	2	4	0	0	0	10	0	0
MA32	1866	9,075,647	5	1	2	1	0	1	10	0	0
MA33	1839	9,076,987	3	0	4	0	0	1	8	1	0
MA34	1842	9,074,856	6	0	6	0	0	2	14	1	0
MA35	1866	9,077,452	1	0	4	0	0	1	6	0	0
MA36	1842	9,085,266	5	0	2	0	0	1	8	0	1
MA37	1842	9,075,909	2	1	3	0	0	0	6	0	0
MA38	1866	9,076,117	5	0	1	0	0	2	8	1	0
MA39	1866	9,077,937	4	2	1	0	0	0	7	1	1
MA40	1842	9,075,612	8	0	3	0	0	2	13	1	0
MA41	1866	9,085,251	3	0	3	0	0	1	7	1	1
MA42	1866	9,076,063	8	0	1	0	0	5	14	2	1

MA43	1866	9,074,510	4	0	1	0	0	3	8	0	0
MA44	1866	9,076,783	2	0	1	0	0	0	3	0	1
MA46	1818	9,075,663	7	1	1	0	1	1	11	1	0
MA47	1866	9,076,535	7	0	2	0	0	0	9	0	0
MA49	1866	9,078,022	9	0	2	0	0	2	13	1	0
Total	85,323	-	229	20	109	3	2	65	428	22	17
Average	1,855	9,080,403	4.98	0.43	2.37	0.07	0.04	1.41	9.3	0.48	0.37

[1] Discrepancies in generation times result from slight differences in numbers of transfer cycles and days incubated until visible colonies appeared. [2] Total number of sites covered by resequenced *Illumina* reads, where the original, ancestral size of the reference genome (DK1622) contains 9,139,763bp. MA, mutation accumulation; SNV, single nucleotide variant; InDel, insertion or deletion.

Supplementary Table 4: Counts of both mutated and total number of sites for all mutational spectra and categories across MA lines relative to reference genome *M. xanthus* DK1622.

[Attached as separate original file: Wielgoss_GBE_2024_TableS4.xlsx]

Supplementary Table 5: Counts of Indel mutations.

Indel category	Total Count	Insertion	Deletion	Fraction coding
Non-STR (Total)	13	2	11	100%
STR (Total)	26	20	6	46%
1-nucleotide STR motifs:				
[C] and [G]	18	15	3	50%
[A] and [T]	5	4	1	20%
3-nucleotide STR motif: [ATG]	1	1	0	100%
5-nucleotide STR motif: [GCCGG]	1	0	1	100%
16-nucleotide STR motif: [CAGAGCCTCGAAATCA]	1	1	0	100%

Supplementary Table 6: Statistical summary of performed dispersion test on mutational count data for single and total base-pair change categories, respectively. Probabilities above 0.05 ($P > 0.05$) indicate that the null hypothesis that count data is Poisson-distributed cannot be refuted.

Mutational category	Sample size	Mean	Variance	***P*(Poisson)**
AT:GC	46	1.15	0.976	0.488
GC:AT	46	3.33	4.05	0.303
AT:TA	46	0.370	0.371	0.924
GC:TA	46	2.72	2.92	0.680
AT:CG	46	1.02	1.18	0.448
GC:CG	46	0.717	0.829	0.438
TOTAL	46	9.30	11.7	0.226
